# Boundary Conditions for Heat Transfer and Evaporative Cooling in the Trachea and Air Sac System of the Domestic Fowl: A Two-Dimensional CFD Analysis

**DOI:** 10.1371/journal.pone.0045315

**Published:** 2012-09-20

**Authors:** Nina S. Sverdlova, Markus Lambertz, Ulrich Witzel, Steven F. Perry

**Affiliations:** 1 Institute for Product and Service Engineering, Ruhr-Universität Bochum, Bochum, Germany; 2 Institute for Zoology, Rheinische Friedrich-Wilhelms-Universität Bonn, Bonn, Germany; Stockholm University, Sweden

## Abstract

Various parts of the respiratory system play an important role in temperature control in birds. We create a simplified computational fluid dynamics (CFD) model of heat exchange in the trachea and air sacs of the domestic fowl (*Gallus domesticus*) in order to investigate the boundary conditions for the convective and evaporative cooling in these parts of the respiratory system. The model is based upon published values for respiratory times, pressures and volumes and upon anatomical data for this species, and the calculated heat exchange is compared with experimentally determined values for the domestic fowl and a closely related, wild species. In addition, we studied the trachea histologically to estimate the thickness of the heat transfer barrier and determine the structure and function of moisture-producing glands. In the transient CFD simulation, the airflow in the trachea of a 2-dimensional model is evoked by changing the volume of the simplified air sac. The heat exchange between the respiratory system and the environment is simulated for different ambient temperatures and humidities, and using two different models of evaporation: constant water vapour concentration model and the droplet injection model. According to the histological results, small mucous glands are numerous but discrete serous glands are lacking on the tracheal surface. The amount of water and heat loss in the simulation is comparable with measured respiratory values previously reported. Tracheal temperature control in the avian respiratory system may be used as a model for extinct or rare animals and could have high relevance for explaining how gigantic, long-necked dinosaurs such as sauropoda might have maintained a high metabolic rate.

## Introduction

Besides supplying oxygen and eliminating carbon dioxide, the respiratory system plays an important role in regulation of body temperature and water balance. The respiratory temperature control is realised through convective and evaporative cooling. The cool air passing through the respiratory system is heated and saturated with water vapour during inspiration. Both processes take place to the large extent in the trachea [1]. During the expiration the energy taken up by the air is transported out of the respiratory system. Some of the water and heat may be resorbed in the nasal cavities [1]. Crawford and Lasiewski [2] calculated an allometric relationship for the total evaporative water loss in nonpasserine birds at an ambient temperature of 25°C. According to Dawson [1], evaporative water loss through the respiratory system varies significantly among avian species and even may be exceeded by the cutaneous water loss at the temperatures below the heat stress.

Computational methods have become a very important tool for modelling and analysing the biology and physiology of extant and extinct animals [3]. In order to use its full potential and obtain good simulations of the physiological processes, the boundary conditions must be precisely set, based on an extant, biological model. A model of the avian respiratory system based on the domestic fowl (*Gallus domesticus*) seems to be very appropriate for this purpose. The anatomy and function of the respiratory system of domestic fowl has been extensively studied experimentally. The gross anatomical and histological structure of the respiratory system in the domestic fowl has been described in detail by several authors [4]–[8]. Airflow parameters and time-dependant pressure change have been determined, and air sac pressure, flow rate and flow volume are available from several experimental studies on the domestic fowl and duck [9].

The evaporative water loss from the respiratory system of the domestic fowl has been measured in normal and heat-stressed conditions by Menuam and Richards [10], and shows a wide variation. A simulation of the evaporative cooling can therefore help to predict a reasonable water loss that corresponds to a given heat loss. Unfortunately, the published data on metabolic heat production and heat loss is based on an overbred species (domestic fowl) and does not necessarily reflect that of a wild, galliform bird. Data for the chukar (*Alectoris chukar*) are available and appropriate because this free-living species is closely related to domestic fowl, both being phasianid galliforms. Frumkin et al. [11] measured heat flux per body weight due to the evaporative cooling in the chukar.

In this study we test the boundary conditions for a computational fluid dynamics (CFD) model of the heat exchange in the avian respiratory system on the example of domestic fowl (*Gallus domesticus*). We create a simplified CFD model of an avian trachea and air sac based on the available anatomical and physiological data. In particular for the simulation of the evaporative cooling in the trachea, along with published data we used our own histological analysis. We gave special attention to the structure of the tracheal glands and to the thickness of the heat conduction barrier between the tracheal surface and the subepithelial capillary network.

Using this information the process of evaporative cooling is simulated. Specifically we address the following questions: 1) what values for the boundary conditions in the computational model lead to fully heated and humidified air; and 2) which model is appropriate for the simulation of the evaporative water supply on the tracheal surface.

## Materials and Methods

### Ethics Statement

The animal material used in the study was obtained in accordance with German animal protection law, § 4, Abs. 1 and 3 of Tierschutzgesetz (Tierschutzgesetz in der Fassung der Bekanntmachung vom 18. Mai 2006 (BGBI. I S. 1206, 1313), last amendment: Artikel 20 des Gesetzes vom 9. Dezember 2010 (BGBI. I S. 1934)).

### Topography of the Model

Since our study focuses on the simulation of evaporative cooling in the trachea, we chose to focus the CFD model accordingly. The structure of the respiratory system in the domestic fowl consists of nasal cavities, a trachea, extrapulmonary bronchi and a complex system of parabronchial lungs connected to a total of 9 air sacs [4]–[6]. In a bird of approximately 2 kg, the partially ossified, cartilage-reinforced trachea has diameter of approximately 6–7 mm over the length of about 160–175 mm [6], [12] and the maximal volume of air sacs is about 350 ml [4], [5].

**Figure 1:**
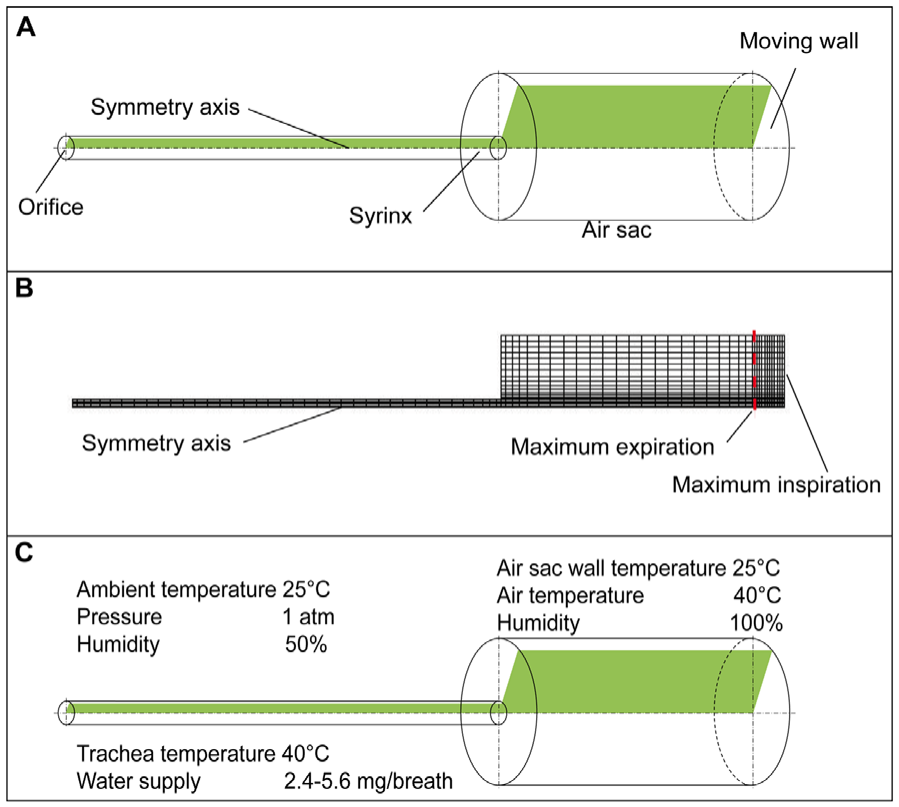
A simplification of the avian respiratory system for the domestic fowl. A) schematic representation; B) 2D finite element model; C) boundary conditions for the 2D model.

The computational model of the respiratory system is reduced to the trachea and air sac with geometrical size based on the anatomical information (Table 1). Accordingly, our model consists of two cylinders (Fig. 1A) representing trachea of 5.8 mm in diameter and 171 mm in length reported in [12] and an air sac dimensioned to concur with published values for the total air sac volume of the fowl respiratory system [4], [5]. This model is represented by an axisymmetric formulation and consists of plane elements. The 2D finite element model of the avian respiratory system (Fig. 1B) was generated using ANSYS ICEM CFD (ANSYS Inc.). Except for the moving posterior wall of the air sac, all surfaces of the model are specified as stationary walls and the orifice is defined as an inlet/outlet vent. The numerical accuracy of the simulation is ensured by the iterative convergence of the results in each individual time step. The mesh sensitivity analysis completed for the range of the representative cell sizes between 3 and 0.3 mm indicated oscillatory convergence in individual time steps.

**Table 1:** Anatomical data for the domestic fowl.

Part of the system	Dimension	Reference
Tracheal diameter	5.8 mm	[12]
Tracheal length	171 mm (in situ)	[12]
Tracheal volume	4.56 ml	[12]
Body mass	2.2 kg	[12]
Total respiratory system volume	414 ml	[4]
Total air sac volume	340–350 ml (87.3%)	[4], [5]

### Simulation of the Breathing Cycle

During inspiration, air sacs are expanded by movement of the ribs and sternum. On expiration, the air is forced into the trachea. The air sac volume that increases during the inspiration and decreases during the expiration leads to the correspondingly changing air sac pressure. In the present study, the simulation of the breathing cycle is based on airflow parameters (Table 2) measured in a domestic fowl with mean weight 1900 g [9]. Several other studies cited by Bouverot and Dejours [9], however, show that domestic fowl with body weights ranging between 1.6 and 3.4 kg display no significant difference in tidal volume or breathing frequency.

**Table 2:** Published respiratory parameters for the domestic fowl with mean weight 1900 g from Bouverot and Dejours [9].

Parameter	Value
Tidal volume	30 ml
Frequency	20 min^−1^
Inspiratory time	1 s
Abdominal air sac pressure during inspiration	−400 Pa
Abdominal air sac pressure during expiration	150 Pa
Ambient air temperature	24°C
Body temperature	40–41°C

**Figure 2:**
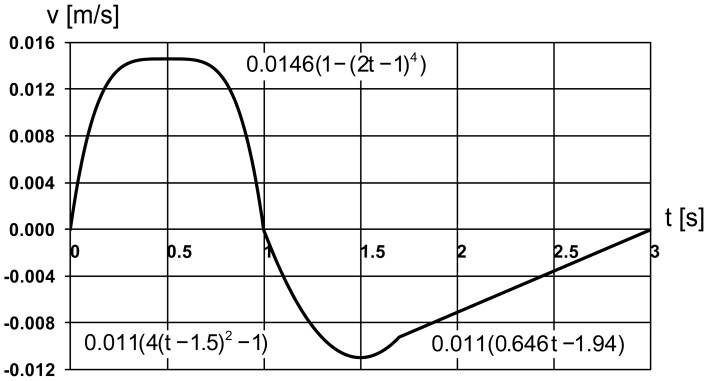
Graph of the moving wall velocity during the breathing cycle used in 2D CFD model.

In the transient simulation of airflow in the trachea is evoked by the movement of the posterior wall of the cylindrical air sac (moving wall in Fig. 1A) guided by a mathematical relationship reflecting a real breathing pattern. Brackenbury [13] showed a very close relationship between the sternum motion and air sac pressure. Thus, we use the data sets on the abdominal air sac pressure change within the breathing cycle [9] in order to define the function for the time dependent motion of the air sac wall. The graph of the air sac wall velocity (Fig. 2) shows a characteristic symmetrical behaviour with plateau during inspiration, which comprises the first third of the three-second breathing cycle, and a peak at the beginning of the expiration with a gradual decrease of the velocity over most of the expiratory phase. This behaviour is modelled by sequential application of three polynomials: one of the 4^th^ power (region with plateau), one of the 2^nd^ power (peak during expiration begin) followed by a 1^st^ power linear decrease:
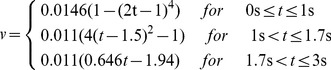
(1)


Note that we normalise the wall motion to fit constraints of fowl average tidal volume and average inspiratory/expiratory times from Table 2 and do not use the measured values of the pressure in air sac directly for the simulation. The measured values of the air sac pressure may be very different depending on the experimental conditions even for voluntarily breathing, unrestrained birds (compare the studies [9] and [13], where as much as two fold difference of pressure values were obtained). The inspiration and expiration are simulated by increasing or decreasing the volume of the air sac by 30 ml sliding the posterior wall of the air sac cylinder along the symmetry axis as shown in Fig. 1B.

The 2D transient simulations were performed in ANSYS FLUENT 12.0.16 and Microsoft Visual Studio 2010 as a C-compiler. We used a user-defined function in order to supply the time dependence for the dynamic mesh with wall velocity (Eq.1). The boundary conditions for the simulation are summarised in Fig. 1C. The ambient pressure is set to atmospheric pressure of 101325 Pa. Even though the air is heated and humidified in the nasal region not represented in the model, the extent of the heating and humidification of air in bird nasal airways is not known. Therefore, in the 2D model the air is assumed to reach the trachea at ambient conditions. The initial conditions of the transient simulation at the initial time of 0 s are: temperature of the air is 40°C, relative humidity 100% due to saturation of the air. Pressure in the air sacs as well as the velocity and temperature of the air in trachea is calculated based on the air sac wall motion.

### Water Loss

The evaporative water loss from the respiratory system of the domestic fowl was previously measured in normal and heat-stressed conditions by Menuam and Richards [10]. The domestic fowls used in their study weighed between 2100 and 2650 g, and were 10–16 months old. These authors observed a highly variable rate of water loss from respiratory system during breathing at rest, amounting to 1.2 mgH_2_O*h^−1^ per g body weight, which corresponds to the water loss in a 2375-g fowl of 47.5 mgH_2_O* min^−1^ or 2.4 mg H_2_O per breath. Similar values of water loss 7.85 mgH_2_O* min^−1^ (or 1.1 mg H_2_O* h^−1^ per g body weight) were measured in chukars with average body mass of 412 g [11]. According to the allometric relationship from the exhaustive review by Crawford and Lasiewski [2] the total evaporative water loss in nonpasserine birds at the ambient temperature of 25°C can be calculated:
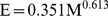
(2)where M is the body mass in gram, E is the evaporative water loss in gram H_2_O per day (24 h). The cited data for domestic fowl of 1650–2750 g ranges between 32 und 55 g H_2_O* day^−1^ of total evaporative water loss, corresponding to 22–38 mg H_2_O *min^−1^. These values include cutaneous water loss as well and, thus, predict much lower respiratory water loss than the study by Menuam and Richards [10]. The available evaporative water loss data are summarised in Table 3.

**Table 3:** Published values for respiratory water and heat loss in the domestic fowl.

Kind of water loss	Value	Heat loss	Reference
Respiratory system, fowl (2100–2650 g)	1.2 mg H_2_Og^−1^ hr^−1^		[10]
Based on [10], converted to 2375 g	2.4 mgH_2_O breath^−1^		
Total evaporative, fowl (1650–2750 g)	32–55 g H_2_O day^−1^		[2]
Calculated for 2375 g based on Eq.2 [2]	1.42 mg H_2_O breath^−1^		
Total evaporative, chukars (412 g)	7.8 mg H_2_O min^−1^	0.77 mJs^−1^ g^−1^	[11]
Based on [11], converted to 2375 g	2.2 mg H_2_O breath^−1^	1.8 Js^−1^ (156 kJ day^−1^)	

FLUENT provides a tool for modelling of gas mixtures and evaporative cooling, and dynamic air properties can be explicitly determined based on the water content in the air. The fluid was modelled as a mixture of air and water vapour with water content based on the Mollier diagram [14]: for example, ambient air at 25°C and 50% humidity contains 0.01 kg H_2_O/kg air, the air in the air sac is considered saturated with water content about 0.48 kg H_2_O/kg.

For humidification of the air passing through trachea, two evaporative models were used. In the first model, a constant water vapour supply was provided on the tracheal surface up to full saturation of air at the syrinx region. Two different runs were performed one with moist (50% humidity) and one with dry (0% humidity) ambient air. In this case the heat flux due to the evaporation is calculated based on the amount of water transported out of the system. In the second model, liquid water droplets were injected into the airways through the trachea surface and only ambient relative humidity of 50% was considered. The upper boundary target value of 2.4 mg H_2_O/breath, which is the maximal value of the water measured in the domestic fowl (Table 3), may lead to incomplete evaporation of the water droplets in the trachea. In the second simulated transient process, the heat flux through the trachea wall is explicitly calculated and the amount water transported out of the system was calculated. For the 2D model we orient on the heat flux of 1.8 J/s due to the evaporative cooling converted from values measured for chukars (Table 3).

### Histology

In order to test the active secretion (injection) model onto the tracheal surface, we investigated the trachea histologically. To this end samples from five different positions along the course of the trachea of two hens and one rooster were harvested from freshly killed animals. The domestic fowls were anesthetised by inhalation of chloroform and killed by transsection of the cervical spinal cord, a method that keeps suffering of the animal to an absolute minimum. This method is permitted according to § 4, Abs. 1 and 3 of German animal protection law (Tierschutzgesetz). The trachea samples were fixed in phosphate-buffered, 4% paraformaldehyde, dehydrated through graded alcohols and embedded in methyl methacrylate (Technovit 7100, Heraeus Kulzer GmbH & Co. KG, 61273 Wehrheim, Germany). Sections of 2 or 3 µm thickness, obtained with a motorised rotation microtome (Microm HM 350, Microm International GmbH, Walldorf, Germany), were stained with 0.1% toluidine blue for about 60 s and analysed using a Leitz DMRBE research microscope.

## Results

**Figure 3:**
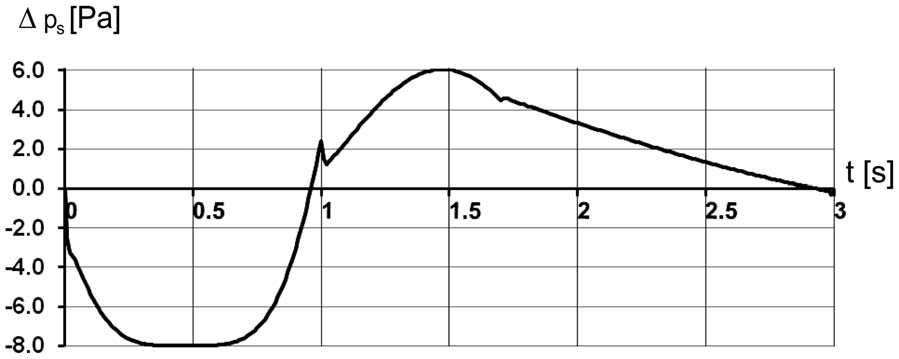
Pressure change in the air sac during one breathing cycle. 1 s corresponds to end-inspiration.

**Figure 4:**
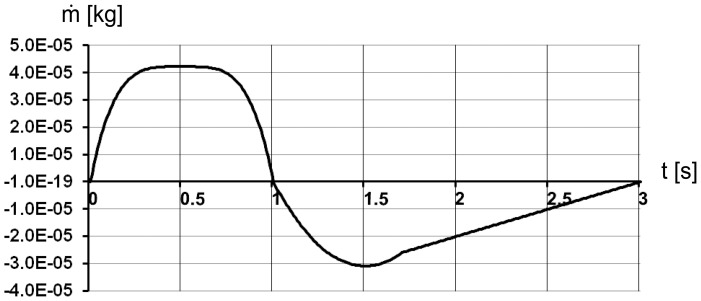
Mass flow through the orifice during one breathing cycle. 1 s corresponds to end-inspiration.

**Figure 5:**
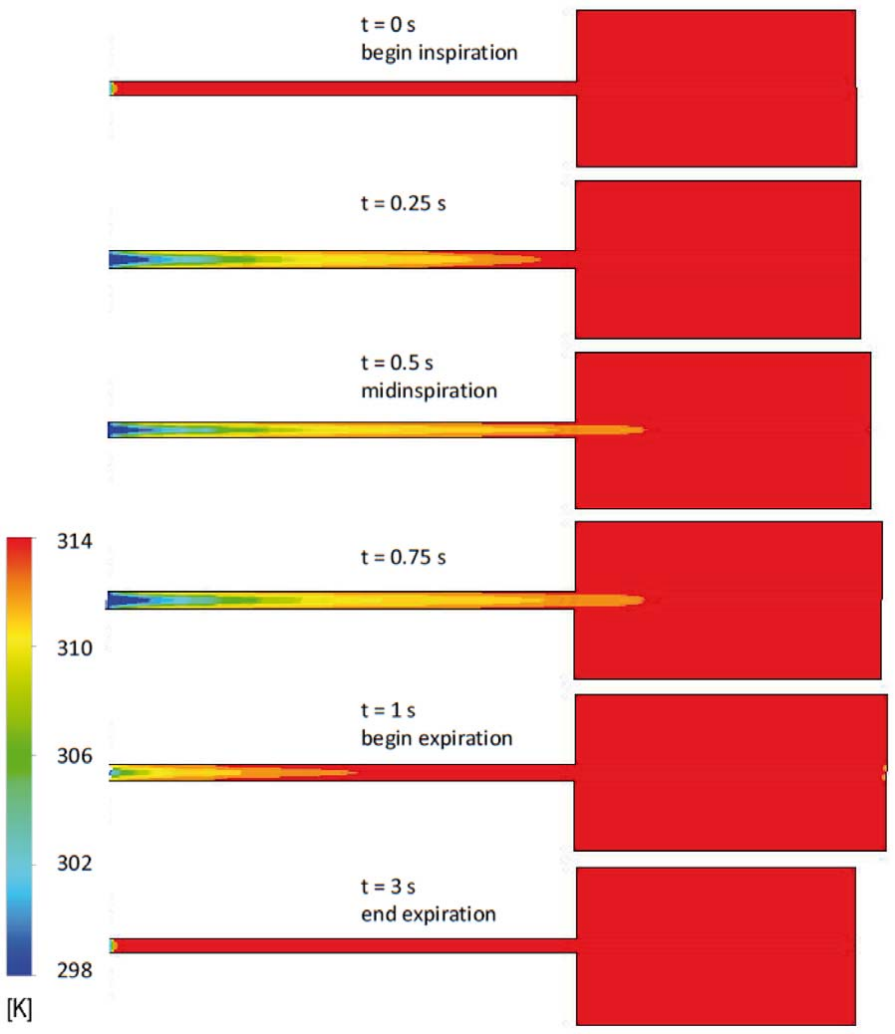
Temperature distribution in the 2D model during the breathing cycle. The maximal volume increase is seen at 1 s at the end of inspiration/begin of expiration, the initial air sac volume is reached at 3 s, end expiration.

**Figure 6:**
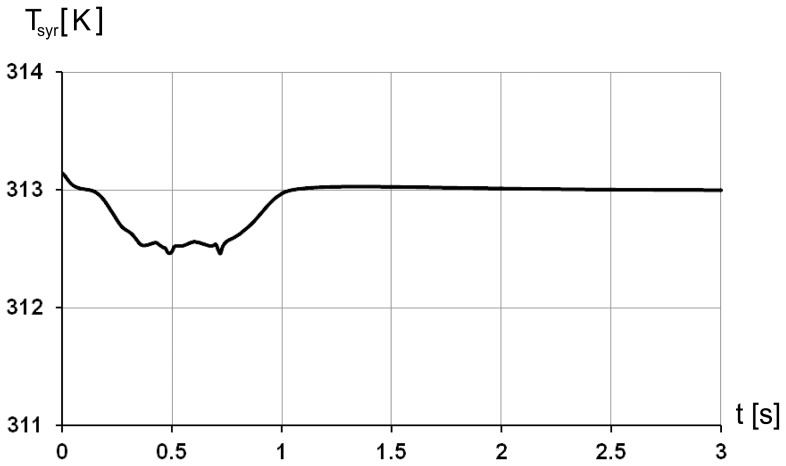
Temperature in syrinx averaged over the cross-sectional area during the breathing cycle. 1 s corresponds to end-inspiration.

The change in air sac pressure relative to the ambient pressure is shown in Fig. 3 and follows closely the profile of the air sac wall velocity (Fig. 2). The negative relative pressure in air sac is the greatest with the value of –8 Pa in the middle of the inspiration. The positive pressure is at maximum of 6 Pa at 1.5 s. The low values of pressure loss are due to the simplified geometry of the model. Mass flow through the orifice is shown in Fig. 4 and totals to 33.3 10^−6^ kg during inhalation and 33.2 10^−6^ kg during exhalation, with corresponds to the volume flow of 29 ml during inspiration and 30.7 ml during expiration. The average air velocity is greatest passing through the orifice, with maximum of 1.4 m/s at 0.5 s during the inspiration and 1.1 m/s at 1.5 s during the expiration. The Reynolds number is below 500 during the inspiration and about 400 during expiration. The air temperature in the trachea is shown in Fig. 5. In the beginning of the breathing cycle the trachea is filled with warm air at the body temperature of 40°C (313 K). The coming in air at 25°C (298 K) is warmed up in the trachea during inspiration almost to the body temperature with the lowest temperature of 39.5°C (312.5 K) in the syrinx during midinspiration at 0.5 s when the highest air flow velocity is reached (Fig. 6). During expiration the warm air from the air sac fills the trachea and the temperature in the air sac remains at 40°C (313 K).

**Figure 7:**
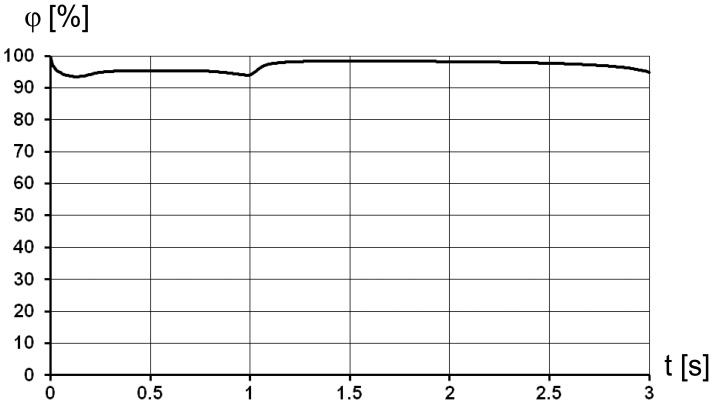
Relative humidity of the air passing through the syrinx in one breathing cycle. 1 s corresponds to end-inspiration.

**Figure 8:**
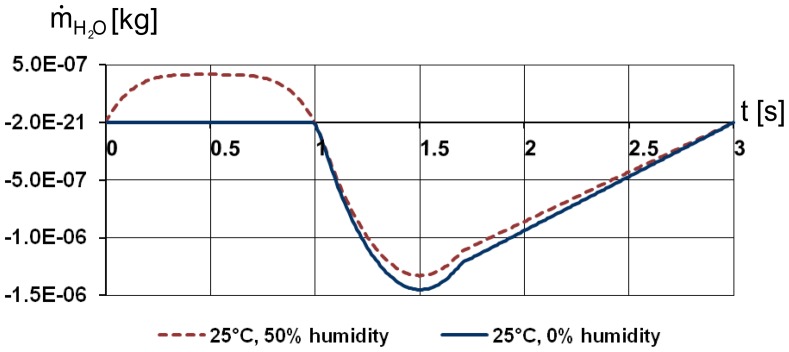
Flow rate of water vapour at tracheal orifice under two simulated experimental conditions. 1 s corresponds to end inspiration.

In the first simulation a constant water vapour concentration was supplied to the tracheal wall in order to obtain water loss and air humidity at the end of the trachea during the breathing cycle. The results show that the air humidity reaches 95% in the syrinx during inspiration and 98.4% during expiration (Fig. 7).The mass flow of water vapour is shown in Fig. 8 for two settings of ambient air humidity. Initially the boundary conditions for ambient air were set at 50% humidity (red dashed line in Fig. 8). The net water loss amounted to 1.09 mg H_2_O/breath and was calculated as the difference between the integral water loss during the expiration phase (1–3 s) 1.42 mg H_2_O and the water influx during the inspiration phase (0–1 s) 0.33 mg H_2_O. The boundary conditions of zero humidity in the ambient air (blue line in Fig. 8) render a net water loss of 1.55 mg H_2_O/breath. As seen in Fig. 8, the water influx is zero during the inspiration.

Thus, the 2D transient simulation showed that the air passing through the trachea is almost fully humidified and warmed almost to core temperature of 40°C by the time it arrives at the syrinx. Consequently, heat exchange takes place almost exclusively in the trachea as a result of heat loss due to warming of the incoming air and evaporative heat loss. The simulation of the convective heat from the tracheal surface results in 0.492 J/breath, whereby the heat transfer takes place during the inspiration phase. This value is below the heat of 0.496 J theoretically required to warm up the inhaled amount of dry air to the body temperature. The evaporative heat loss is calculated based on the amount of water loss using the enthalpy of vaporization of water [15]. Assuming that the secreted water is at about body temperature of 40°C, the value of the enthalpy of vaporization is 2.406 J/mg. This is the lowest estimate of the value, for the cooled tracheal surfaces the value may increase by 1.5% at the most. Thus, for ambient relative humidity of 50% and 0%, the evaporative heat loss amounts to 2.6 and 3.7 J/breath or 76 and 107 kJ/day, corresponding to the water loss of 1.09 and 1.55 mg H_2_O/breath. The total heat loss then amounts to 3.1 and 4.2 J/breath or 90 and 122 kJ/day, respectively.

In the second simulation we use the model of injection of water droplets from the trachea surface at the measured respiratory water rates. In order to correctly apply this simulation, histological analysis of the trachea was performed.

**Figure 9:**
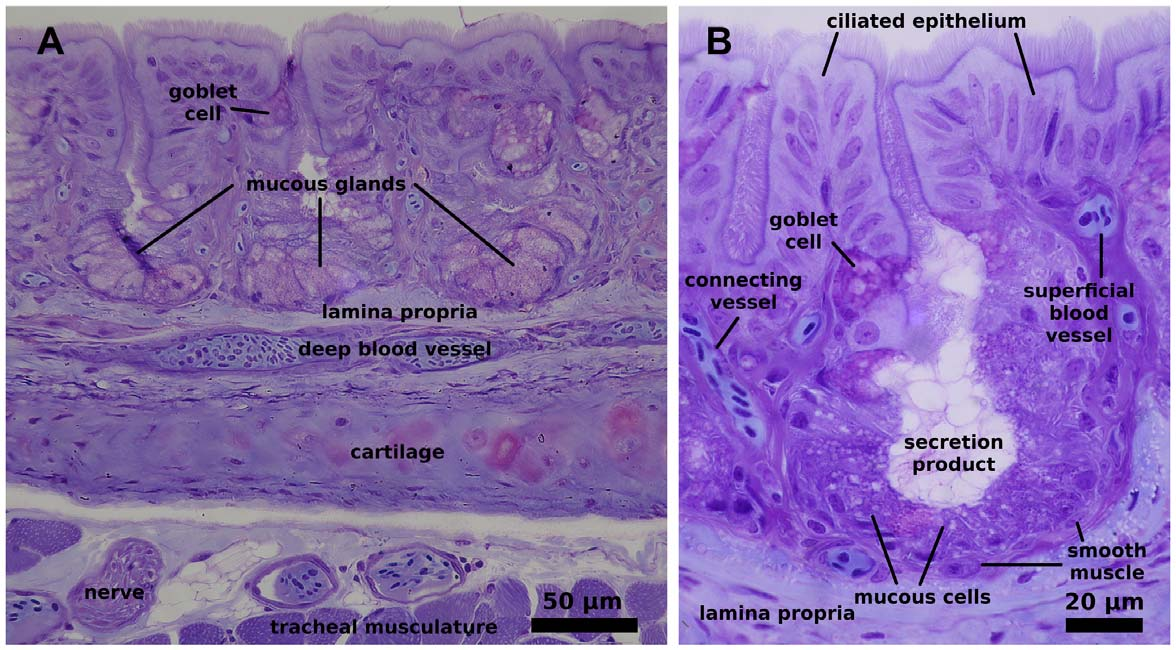
Histological structure of the chicken trachea. A) overview illustrating mucosa with alveolar mucous glands. Note interstitial connective tissue (lamina propria) between the glands contains many blood vessel profiles. The deep vascular layer lies between the tracheal cartilage and the mucosa. B) detail of an alveolar mucous gland showing goblet cells (only one indicated) at its entrance. Note the proximity of the most superficial blood vessels to the luminal surface of the ciliated epithelium. Scale indicated by bars. Staining of 2 µm thick sections with 0.1% toluidine blue.

Histologically the trachea of the domestic fowl corresponds to what is described for birds in general [7], [8]. It is lined by ciliated columnar epithelium which contains numerous, simple alveolar glands (Fig. 9). The glands contain mucous cells, which often present a granular appearance. Numerous alveolar tracheal glands are present up to the distal region of the trachea rather than being replaced there by goblet cells.

The alveolar lamina propria, which composes the extraepithelial part of the heat transfer barrier, consists primarily of dense, irregular connective tissue and contains some smooth muscle and numerous small blood vessels. A network of large blood vessels within the lamina propria encircles the epithelial layer and gives rise to smaller vessels, which ascend between the alveolar glands. These connecting vessels are convoluted and do not suggest countercurrent function. The entire subepithelial region is well vascularised and no obvious differences were observed between the anterior and posterior tracheal regions. The resulting finest vessels come to lie immediately beneath the epithelial cells. Orthogonal distances between the larger and deeper vessels and the tracheal lumen average about 120 µm; whereas the smaller vessels are about 50 µm below the tracheal surface, but frequently even only 30 µm. There is no indication of systematic changes in these dimensions along the length of the trachea.

Our histological results are consistent with the CFD model with droplet injection from the tracheal surface applies only if droplet velocity is set to zero, resulting in a continuous and equally distributed layer of mucous. We found that a higher mass rate of liquid water supply to the trachea is needed to meet the full humidification of the air by the time it reaches syrinx than the measured value (Table 3), which was initially assumed as the upper limit for the analysis. This is due to the incomplete vaporisation of the water droplets during the simulation. However, the pattern of the humidity level in syrinx remains the almost same as in Fig. 7. When mass flow rate of water is set to 4.4 mg/breath the minimal relative humidity level during inspiration is at about 80%, and when mass flow rate of water is set to 5.6 mg/breath the minimal relative humidity level during inspiration is at about 98%. Due to the uniform water supply along the trachea, the humidity values reach local peaks at the beginning and at the end of the inspiration phase, when the flow velocity approaches zero. The mass flow rate of water vapour follows the same pattern as in Fig. 8. The water loss calculated from the simulation data is slightly higher than in constant water vapour supply simulation and sums up to 1.16 mg H_2_O for 4.4 mg/breath water supply on the tracheal surface and to 1.19 mg H_2_O for 5.6 mg/breath water supply.

## Discussion

In the present study we simulate flow through the trachea by changing air sac volume during breathing. Within simulated boundary conditions we study the heat exchange between the tracheal surface and air flowing through the trachea in the computational model. The morphology of the 2D model is based on the available anatomical information [4]–[8], [12] and the boundary conditions using available physiological data for domestic fowl [9]–[11], [13]. Our limited sample suggested that the trachea may be narrower distally than proximally as one author has documented [16], but other investigators did not notice this and we do not feel justified in deviating from a more generalised, uniform tubular model. We assume the humidification of the air by the water supplied on the tracheal surface, which corresponds to the presence of the mucous glands in trachea. It is unlikely that the thin epithelium of air sacs [6] significantly contributes to the humidification of the air. Since the histological structure did not reveal any obvious structural differences along the length of the trachea, the surface properties of the trachea were approximated by constant values. The transient process of breathing is simulated using the moving air sac wall, the velocity of which is based on the air sac pressure change measured in vivo [13].

The air flow parameters based on the tidal volume data [9] produce very low pressure differential values in our model compared with the measured air sac pressures. This is attributed to the simplified geometry of the system and corresponds to the theoretical pressure loss value for this geometry. A 3D model presently being developed more closely reflects the geometry of the fowl airways and may produce better correlation to the pressure drop level in the air sac than obtained in the present study. However, the air flow velocity and heat exchange parameters are in good agreement with the experimental ones. In particular, the model shows that air is warmed and humidified in the trachea rather than in the air sacs. In the first (evaporative) model, a constant water vapour concentration is supplied to the tracheal surface. In the second model, water droplets are supplied to the tracheal surface at the rate higher than initially assumed based on the measurements, but not all supplied liquid is evaporated. The model with constant water vapour supply on the tracheal surface works very well, but delivers the heat loss based on the water loss in the system that is somewhat lower than measured experimentally. The droplet injection model produces slightly higher water loss in the system than in the first simulation, but leads to very high relative humidity values in the syrinx at low air flow velocities. These possible artefacts suggest non-uniform conditions along the trachea. A more sophisticated model with time-dependent and/or non-uniform water supply along the trachea may better simulate the true condition. The findings of Menuam and Richards [10] show different water loss values for upper and lower airways.

Comparison of the present calculated values for water and heat loss with published ones shows that predictions based on our model lie within realistic limits, in spite of the simplified conditions. In both simulations, the water loss is virtually the same and for the comparison we calculate heat loss based on the heat needed to vaporise the water lost in the system. Experimentally, evaporative heat loss [11] is also based on the measured water loss and obtained in the same way. The calculated water loss is higher for the boundary conditions of the dry air (zero humidity) than for the ambient air with 50% relative humidity. This correlates well with Crawfold and Lasiewski [2], who reported a higher evaporative water loss for lower ambient water vapour pressure. Note that although experimental conditions employ dry or dehydrated air, the evaporative water loss calculated in our simulation is still 25–50% lower than that measured experimentally in the chukar [11]. This could be due to the reduction of our model to trachea and air sac and to the lack of glottis, mouth, and nasal regions, which contribute significantly to the evaporative cooling. In addition, whereas the domestic fowl is bred for large muscle mass or egg production, the chukar is a wild partridge that inhabits semi-arid biotopes [11] is therefore to be expected that the evaporative cooling values per unit body mass would be less in the domestic fowl than its wild relative.

Despite the simplifications, the present 2D model produces plausible results and has an advantage of requiring much less computing time than a 3D model. This allows rapid testing of different boundary conditions.

Given the ultimate goal of the study, namely the application of the present model to study the heat exchange strategies in an extinct animal group [17]–[19], it is encouraging that a relatively simple model can produce realistic results. In other words, the role of a wet, tracheal surface in temperature control follows mathematically from the basic geometry of a long tube, a total air volume that is much larger than the tidal volume, and the physical properties of water and soft tissue are probably similar across species. Just as extinct animals are inaccessible, in human and veterinary medicine direct experimentation is not possible, the present model could also find application in areas such as anaesthesiology, in respirator design or in treatment of exotic zoo animals.

### Conclusions

We conclude that the present CFD model is able to reliably predict the properties of end-expired and inspired air based on the geometrical and physical properties of the tracheal surface, and employing measured values for tidal volume and air sac pressures in the domestic fowl. Applications of the present model to extinct animals or to veterinary medicine are implicit.
